# Simulation-based ultrasound-guided lumbar puncture training using a flipped classroom: a single-arm pilot programme evaluation

**DOI:** 10.1186/s12909-026-10070-8

**Published:** 2026-08-01

**Authors:** Chao-Yen Huang, Sai-Wai Ho, Po-Ming Chen, Chia-Ching Chen

**Affiliations:** 1 School of Medicine, Chung Shan Medical University, Taichung, Taiwan, ROC; 2 Emergency Department, Chung Shan Medical University Hospital, Taichung, Taiwan, ROC; 3 Research Assistant Center, Show Chwan Memorial Hospital, Changhua, Taiwan, ROC; 4 Department of Nursing, Central Taiwan University of Science and Technology, Taichung, Taiwan, ROC; 5 Institute for Research into Cancer of the Digestive System (IRCAD) - Taiwan and Asian Institute of TeleSurgery, Show Chwan Health Care System, Changhua, Taiwan, ROC; 6 Department of Emergency Medicine, Chang Bing Show Chwan Memorial Hospital, No. 6 Lugong Road. Lukang Zhen, Changhua, Taiwan, ROC

**Keywords:** Simulation-based education, Ultrasound, Lumbar puncture, Flipped classroom, Pilot study, Emergency medicine, Medical education

## Abstract

**Background:**

Traditional landmark-guided lumbar puncture (LP) has high failure rates among novice emergency medicine (EM) clinicians. While ultrasound guidance improves success, structured training models remain limited and expensive. We evaluated a cost-effective, simulation-based flipped classroom curriculum for teaching ultrasound-guided LP to EM residents and trainees.

**Methods:**

We developed a high-fidelity, custom 3D-printed spinal simulator costing approximately $40 USD per unit. The curriculum utilized a flipped classroom model combining online theoretical modules with hands-on, deliberate practice.

**Results:**

Twenty-two participants (9 EM residents, 13 PGY trainees) completed the training programme; paired landmark-versus-ultrasound OSCE data were available for the seven residents who completed both phases. Among EM residents with paired data (n = 7), the ultrasound-guided phase OSCE completion rate was 100%, compared to 43% during the earlier landmark phase (a large but statistically non-significant difference, p = 0.125, McNemar’s exact test). The ultrasound-guided phase was associated with higher rubric-rated performance scores and overall ratings (nominally significant, uncorrected for multiple comparisons); needle passes (median 7 vs. 12) and procedure times were also lower but did not reach statistical significance in this small paired sample. Both groups reported significantly lower procedural stress with ultrasound guidance, with a smaller, non-significant increase in confidence.

**Conclusions:**

A flipped classroom curriculum utilizing a low-cost, 3D-printed simulator is a feasible and well-received educational innovation for teaching ultrasound-guided LP, with encouraging but preliminary performance findings for EM residents that require confirmation in controlled studies.

**Supplementary Information:**

The online version contains supplementary material available at https://doi.org/10.1186/s12909-026-10070-8.

## Introduction

Lumbar puncture (LP) remains a fundamental procedure for diagnosing central nervous system infections and neurological emergencies [1]. Traditional landmark-guided LP relies on palpation of anatomical structures, a technique with 30–71% error rates in identifying the correct intervertebral spaces [2, 3]. Obesity, anatomical variations, and positioning difficulties compound these challenges, resulting in multiple puncture attempts, failed procedures, and complications including post-dural puncture headache and traumatic taps [4, 5].

Ultrasound guidance improves LP success rates and reduces complications through real-time visualisation of anatomical structures [6]. A systematic review by Shaikh et al. [6] showed that ultrasound-assisted LP reduced traumatic tap risk, decreased puncture attempts, and improved first-attempt success rates; a randomised trial in an unselected emergency-department population, however, found no overall advantage to routine ultrasound localisation [7], suggesting that any benefit may be greatest in patients who are difficult to palpate or obese. However, to our knowledge, formal ultrasound-guided LP training is not yet widely incorporated into medical curricula in Taiwan, creating a knowledge gap that this study sought to address. Internationally, ultrasound guidance for LP is increasingly recommended (recent systematic reviews report improved first-attempt success and fewer traumatic taps, particularly in children and when the operator is less experienced [8]), yet structured, simulation-based training with validated, low-cost models remains unevenly adopted across settings. Furthermore, recent studies emphasizing simulation-based ultrasound-guided LP have reported its value for comparing and practising different real-time techniques [9].

Two theoretical frameworks underpin this educational intervention. First, Kolb’s experiential learning theory [10] posits that effective learning occurs through a cycle of concrete experience, reflective observation, abstract conceptualisation, and active experimentation, providing a rationale for hands-on simulation training following theoretical preparation. Second, deliberate practice theory emphasises repetitive skill development with structured feedback as the primary mechanism for achieving expert performance [11]. High-fidelity simulation addresses the ethical tension between trainee learning and patient safety by enabling mistake-based learning without patient harm [12].

The flipped classroom model, where learners complete theoretical content before hands-on practice, has shown promising results in health-professions education [13]. This approach maximises instructor-led session efficiency by dedicating time to deliberate practice rather than passive knowledge transmission.

We evaluated whether simulation-based education using a flipped classroom model could effectively teach ultrasound-guided LP. Our study employed a two-tier evaluation approach: (1) objective performance assessment through OSCE in EM residents, and (2) training experience evaluation including psychological measures and satisfaction across all participants. We hypothesised that ultrasound-guided training would produce superior OSCE performance in residents and that all trainees would report reduced procedural stress and increased confidence compared to traditional landmark techniques.

## Methods

### Study design and setting

This prospective programme evaluation was conducted at Chung Shan Medical University Hospital, a tertiary medical centre in central Taiwan, from January to June 2022. The simulation-based LP training programme represents the standard curriculum in the EM department. The Institutional Review Board of Chung Shan Medical University Hospital granted exemption from full review (Exemption No. CS2-25220), as the educational intervention was part of the routine curriculum and data were collected as part of standard programme evaluation. EM residents participated as part of mandatory curriculum requirements; post-graduate year (PGY) trainees participated voluntarily, with assurance that non-participation would not affect clinical evaluations. All participants provided informed consent for educational participation and for publication of anonymised aggregate outcomes. This study is reported in accordance with the Strengthening the Reporting of Observational Studies in Epidemiology (STROBE) statement [14]. A completed STROBE checklist is provided as Additional file 1.

### Participants and flow

**Fig. 1:**
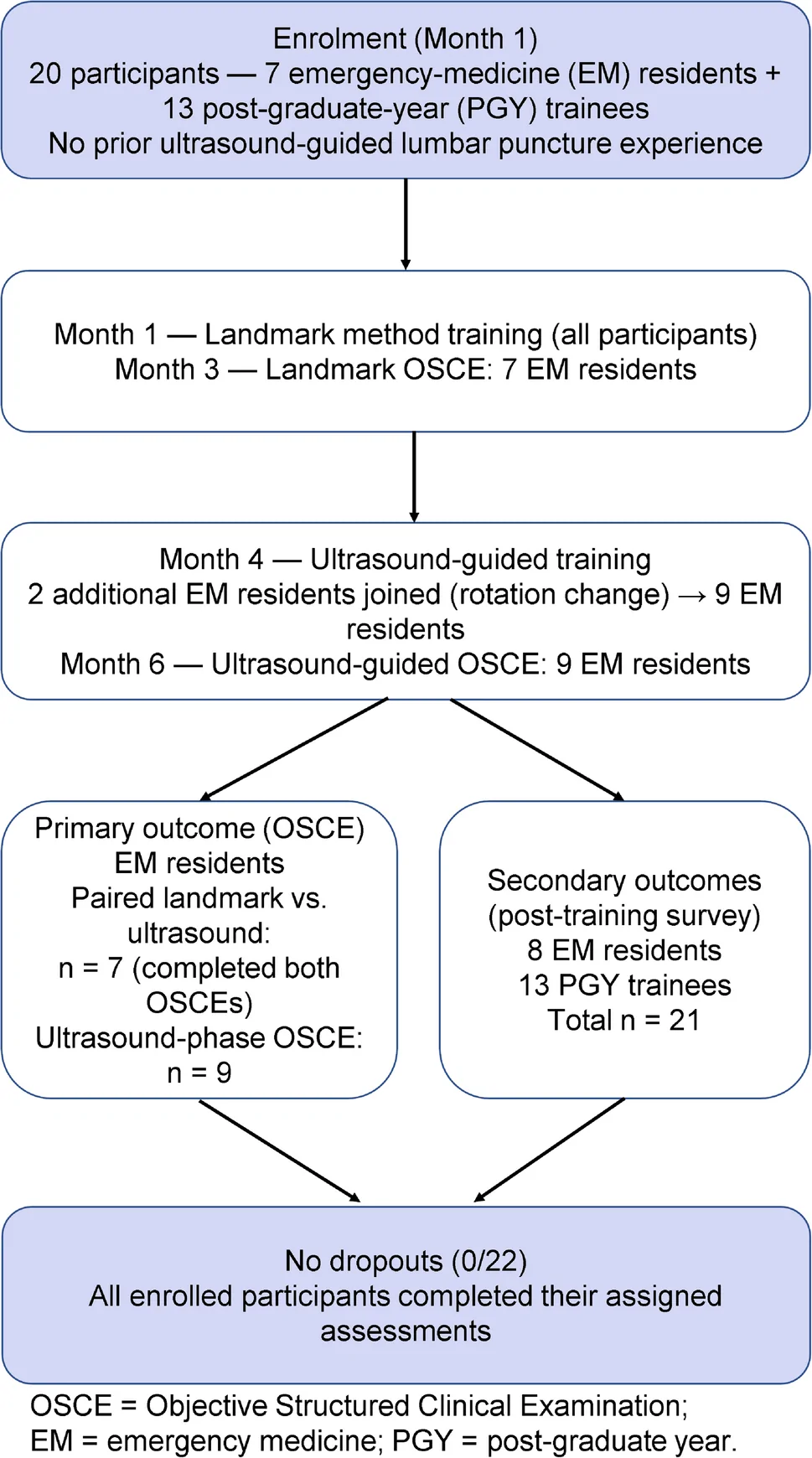
Participant flow diagram. Initial enrolment consisted of 20 participants (7 EM residents, 13 PGY trainees) with no prior ultrasound‑guided lumbar puncture experience. Two additional EM residents joined during the study due to rotation changes. Training and assessments were conducted at months 1, 3, 4, and 6 as shown. Landmark OSCE was completed by 7 residents; US‑guided OSCE was completed by 9 residents. Primary outcome analysis used paired data from the 7 residents who completed both OSCEs. Secondary outcomes included 8 EM residents (survey) and all 21 participants (8 EM + 13 PGY). No dropouts occurred. OSCE = Objective Structured Clinical Examination; US = ultrasound; LP = lumbar puncture; PGY = post‑graduate year

Twenty-two participants were enrolled across two groups (Fig. 1). Nine emergency-medicine residents took part (seven from the outset and two who joined for the ultrasound phase), each with 1–4 years of clinical experience (mean 2.4 years) and 5–20 previous landmark-guided lumbar punctures; their participation was mandatory, and no eligible resident declined. The remaining 13 participants were post-graduate-year (PGY) trainees in their first clinical year, each with minimal prior lumbar-puncture experience (0–2 procedures), who participated voluntarily, which may have introduced selection bias. Critically, all participants (100%) had no prior experience with ultrasound-guided lumbar puncture.

All seven initial EM residents completed landmark training (Month 1) and landmark OSCE (Month 3). Two additional EM residents joined the ultrasound training phase (Month 4) due to rotation schedule changes, resulting in nine residents completing the ultrasound OSCE (Month 6). These two additional residents completed ultrasound training and OSCE but were excluded from paired analyses comparing landmark versus ultrasound performance. Seven residents completed both OSCEs, enabling paired comparisons. One resident was unavailable for psychological surveys, yielding *n* = 8 for secondary outcomes in the resident group. All 13 PGY trainees completed both training phases and psychological assessments but did not undergo OSCE evaluation due to resource constraints (limited OSCE examiner availability and simulation laboratory time). This pragmatic decision represents a limitation. There were no dropouts (Fig. 1). In summary, analytic denominators differed by outcome: paired landmark-versus-ultrasound OSCE comparison, *n* = 7 residents; ultrasound-phase OSCE, *n* = 9 residents; and post-training psychological surveys, *n* = 8 residents and *n* = 13 PGY trainees (21 respondents in total).

### Educational intervention

The training programme was implemented over six months using a flipped classroom model. In this flipped-classroom design, trainees completed each self-paced online pre-learning module before attending the corresponding in-person workshop; a short pre-test confirmed engagement with the online content, and instructor-led time was reserved for hands-on deliberate practice. A full outline of the curriculum and e-learning modules is provided as Additional file 2. Month 1 – Landmark method training: A 15-minute online pre-learning module covering LP indications, contraindications, anatomical landmarks, procedural steps, and complications, followed by a 2-hour hands-on workshop using the Kyoto Kagaku Lumbar Puncture Simulator II M43B (Kyoto Kagaku Co., Kyoto, Japan) with a 1:6 instructor-to-trainee ratio and immediate feedback. Month 3 – Landmark method OSCE: EM residents (*n* = 7) completed standardised OSCE evaluation (8-minute time limit). Month 4 – Ultrasound-guided method training: A 20-minute online pre-learning module covering ultrasound anatomy, image optimisation, target identification, needle guidance techniques, and complication detection, followed by a 2.5-hour hands-on workshop with three stations: (1) pre-procedure ultrasound assessment using portable ultrasound devices (Butterfly iQ, USA; BenQ T3300, Taiwan), (2) ultrasound-guided needle insertion using a custom 3D-printed simulator, and (3) post-procedure assessment and complication detection. The same 1:6 instructor-to-trainee ratio was maintained. Month 6 – Ultrasound-guided method OSCE: EM residents (*n* = 9) completed evaluation (8-minute time limit).

Both OSCEs used an identical 8-minute (480-second) time limit. All successful procedures were completed well within this limit, indicating that the time limit did not differentially constrain either method; the corresponding mean procedure times are reported in the Results (Table 1).

**Table 1:** OSCE performance comparison: Landmark vs. Ultrasound-Guided Methods (Emergency Medicine Residents)

Panel A. Paired Comparison (*n* = 7 residents who completed both assessments)				
**Outcome**	Landmark (*n* = 7)	Ultrasound (*n* = 7)	**p-value**	**Cohen’s dz**
Performance Score (0–12)	4.43 ± 1.68	8.00 ± 1.93	0.021	1.17
Overall Rating (0–5)	1.86 ± 0.83	4.00 ± 0.93	0.015	1.28
Number of Needle Passes	13.29 ± 9.22 (median 12)	5.57 ± 2.13 (median 7)	0.101	0.73
Procedure Time (sec)	315 ± 131	195 ± 100	0.180	0.57
Completion Rate	43% (3/7)	100% (7/7)	0.125†	—

Panel B. Descriptive Statistics — All Ultrasound-Phase Residents (*n* = 9)				
**Outcome**		Ultrasound (*n* = 9)		
Performance Score (0–12)		8.11 ± 1.73		
Overall Rating (0–5)		4.11 ± 0.87		
Number of Needle Passes		5.56 ± 2.01		
Procedure Time (sec)		208 ± 94		
Completion Rate		100% (9/9)		

Data presented as mean ± SD or percentage (n/N). p-values from paired t-tests (*n* = 7). Wilcoxon signed-rank p-values were 0.047 (performance score), 0.047 (overall rating), 0.062 (punctures), and 0.219 (procedure time); effect sizes are Cohen’s dz (paired); medians are shown for puncture count given its skewed distribution. The two nominally significant differences would not remain significant after Bonferroni or Holm correction across the five primary outcomes

Panel A ultrasound values reflect only the 7 residents included in paired analyses

Panel B includes all 9 residents who completed the ultrasound-guided OSCE (7 original + 2 who joined during the ultrasound phase due to rotation changes; excluded from paired comparisons)

†McNemar’s exact test (paired, *n* = 7)

The OSCE scoring rubric was developed by two senior EM faculty members (C.Y.H. and C.C.C.) based on established LP procedural competency frameworks and piloted with two volunteer trainees prior to the study. The same seven-item, 0–2 rubric (maximum 12 points) was used for both OSCEs; the two versions were identical except for the second item, which assessed the localisation step specific to each method (landmark palpation versus ultrasound-guided site identification). All OSCE performances were scored by a single unblinded assessor (C.C.C.), who also delivered the curriculum; because scoring was performed by one rater, inter-rater reliability could not be assessed. That assessor scored each resident’s performance during all OSCE sessions. The method could not be masked because the use of ultrasound equipment was visually apparent. To minimise bias, standardised scoring criteria with objective anchors were used, and primary emphasis was placed on objective outcomes (completion status, needle-pass count, procedure time). Completion was defined as successful aspiration of simulated cerebrospinal fluid within the allotted time limit. Needle passes were counted as each forward advance of the needle, including any redirection after a partial withdrawal without complete removal from the skin (i.e., each withdrawal-and-re-advance was counted). Because this count is finer-grained than the skin-puncture “attempts” reported in some previous studies, the absolute values are not directly comparable across studies, although the within-participant landmark-versus-ultrasound comparison is unaffected. Throughout the training period (Months 2–6), no clinical LP cases were observed and no additional practice sessions were scheduled.

### Custom ultrasound-guided LP simulator

We developed a high-fidelity 3D-printed spinal model following Odom et al.’s [15] methodology. High-resolution CT images were used to create anatomically accurate 3D models of the lumbar spine (T12–sacrum with iliac bones). Custom-formulated silicone materials encased the printed spine, designed to mimic human tissue compliance, provide distinct ultrasound acoustic properties, deliver realistic tactile needle feedback, and maintain durability for repeated use. An 18-French urinary catheter was modified into a closed-loop system within the spinal canal to simulate cerebrospinal fluid (CSF). Five experienced physicians (three EM specialists and two neurologists) reviewed the model and confirmed that its ultrasound appearance and needle-insertion feedback approximated those of a real patient (face validity); formal quantitative validity scoring was not undertaken. The estimated material cost per simulator was approximately USD 40, compared to approximately USD 1,000 for comparable commercial lumbar puncture trainers, representing a potential cost advantage for resource-limited training settings.

### Outcome measures

Primary outcomes (OSCE performance, EM residents): performance score (0–12 points across seven technical items scored 0–2 each: accurate patient positioning, proper needle insertion site, avoidance of repeated punctures causing haematoma, proper needle angle and depth control, correct hand positioning, correct stylet removal and CSF observation, and efficient CSF drainage completion); overall performance rating (0–5 scale); number of needle passes; procedure time (seconds from needle insertion to CSF collection); and completion status (successful CSF collection within time limit).

Secondary outcomes (training experience, all participants): self-reported perceived stress (0–5 scale) and confidence (0–5 scale) for each method, collected once at the end of the programme through a single retrospective questionnaire in which every participant rated both the landmark and the ultrasound method using single-item measures. While validated multi-item instruments exist, single-item measures were selected for practical feasibility and have demonstrated acceptable validity for global constructs in educational research [16]. These are considered exploratory self-report indicators rather than robust psychometric outcomes. Additionally, method preference, perceived advantages (5-point Likert scale), and course satisfaction (5-point Likert scale) were assessed.

### Statistical analysis

Analyses were performed using SPSS version 26.0 (IBM Corp., Armonk, NY). Normality was assessed using Shapiro–Wilk tests (no significant departures were detected, *p* > 0.05); however, given the small sample (*n* = 7), formal normality testing has limited power, so Wilcoxon signed-rank sensitivity analyses were also performed for the primary continuous outcomes. For primary outcomes, paired t-tests compared OSCE performance metrics between landmark and ultrasound methods for the seven residents who completed both assessments. McNemar’s exact test compared the paired completion status of the seven residents who completed both OSCEs (Fisher’s exact test was used for the descriptive all-nine completion comparison). For secondary outcomes, self-reported stress and confidence were compared between the landmark and ultrasound methods within each group (EM residents and PGY trainees) using Wilcoxon signed-rank tests, with 95% confidence intervals for the mean within-participant difference; method preference and course satisfaction are reported descriptively (proportion or mean ± SD). Given the small samples, these analyses are considered exploratory and hypothesis-generating. Effect sizes for the paired comparisons were expressed as Cohen’s dz, the standardised mean of the within-participant differences (small = 0.2, medium = 0.5, large = 0.8). Given the exploratory nature and small sample size, no adjustments were made for multiple comparisons; reported p-values are nominal and should be interpreted accordingly, and neither of the two nominally significant primary outcomes would remain significant after Bonferroni or Holm correction across the five primary outcomes. Statistical significance was set at *p* < 0.05 (two-tailed). Given the small paired sample (*n* = 7), Wilcoxon signed-rank tests were also performed as sensitivity analyses for all primary continuous outcomes; results were directionally concordant with the parametric tests (with the smallest attainable two-sided p at *n* = 7 being ≈ 0.016) and are reported alongside the parametric results in Table 1. No a priori sample-size calculation was performed; participants comprised a convenience sample of all available EM residents and PGY trainees, and the study is therefore framed as exploratory and hypothesis-generating.

## Results

### Primary outcomes: OSCE performance (EM residents)

Nine EM residents completed ultrasound-guided OSCEs; seven completed both landmark and ultrasound assessments, enabling paired comparisons (Fig. 1). Table 1 presents performance data in two panels: Panel A shows paired comparisons for the seven residents who completed both assessments, and Panel B shows descriptive statistics for all nine residents who completed the ultrasound-guided OSCE.

In paired comparisons (*n* = 7, Table 1 Panel A), performance during the ultrasound-guided phase was better than during the earlier landmark phase for the two rated outcomes (nominally significant, uncorrected for multiple comparisons): performance score (8.00 ± 1.93 vs. 4.43 ± 1.68, *p* = 0.021; Wilcoxon *p* = 0.047; Cohen’s dz = 1.17) and overall rating (4.00 ± 0.93 vs. 1.86 ± 0.83, *p* = 0.015; Wilcoxon *p* = 0.047; dz = 1.28). The number of needle passes (median 7 vs. 12; mean 5.57 ± 2.13 vs. 13.29 ± 9.22, *p* = 0.101; Wilcoxon *p* = 0.062) and procedure time (195 ± 100 vs. 315 ± 131 s, *p* = 0.180; Wilcoxon *p* = 0.219) both favoured the ultrasound-guided phase but did not reach statistical significance in this small paired sample. Completion rate increased from 43% (3/7) to 100% (7/7), though this difference did not reach statistical significance in the paired sample (*p* = 0.125, McNemar’s exact test), likely reflecting limited statistical power with *n* = 7. Including all nine residents who completed the ultrasound-guided OSCE (Table 1 Panel B), the completion rate was 100% (9/9).

Moderate-to-large effect sizes (Cohen’s dz 0.57–1.28) suggest potentially meaningful differences, although the sequential design and small sample size preclude definitive causal attribution to ultrasound guidance itself versus accumulated procedural experience.

### Secondary outcomes: training experience (all participants)

EM residents (*n* = 8) reported significantly lower stress with ultrasound guidance (2.38 ± 1.11 vs. 3.50 ± 1.00; Wilcoxon *p* = 0.016), with a non-significant increase in confidence (3.50 ± 1.41 vs. 3.00 ± 0.87; *p* = 0.547); 75% (6/8) preferred ultrasound guidance. PGY trainees (*n* = 13) showed the same pattern: significantly lower stress (3.69 ± 0.61 vs. 4.46 ± 0.63; *p* = 0.014) and a non-significant increase in confidence (2.77 ± 0.80 vs. 2.31 ± 1.20; *p* = 0.250); 54% (7/13) preferred ultrasound guidance (Table 2). PGY trainees consistently reported higher baseline stress and lower confidence than EM residents, reflecting limited clinical experience.

**Table 2:** Psychological measures post-training

Measure	Emergency Residents (*n* = 8)	PGY Trainees (*n* = 13)
Stress (0–5 scale)		
Landmark method	3.50 ± 1.00	4.46 ± 0.63
US-guided method	2.38 ± 1.11	3.69 ± 0.61
Change (US − LM); 95% CI; p	−1.12 (− 1.66 to − 0.59); *p* = 0.016	−0.77 (− 1.21 to − 0.33); *p* = 0.014
Confidence (0–5 scale)		
Landmark method	3.00 ± 0.87	2.31 ± 1.20
US-guided method	3.50 ± 1.41	2.77 ± 0.80
Change (US − LM); 95% CI; p	+ 0.50 (− 0.91 to + 1.91); *p* = 0.547	+ 0.46 (− 0.38 to + 1.30); *p* = 0.250
Method Preference		
Landmark	25% (2/8)	46% (6/13)
Ultrasound-guided	75% (6/8)	54% (7/13)

Data presented as mean ± SD or proportion. Stress and confidence were compared with paired Wilcoxon signed-rank tests; the change row shows the mean within-participant difference (US − landmark) with its 95% confidence interval and the Wilcoxon *P*-value. Method preference is descriptive

Course satisfaction ratings were universally high for ultrasound-guided training (mean 5.0/5.0 across all domains), compared to lower scores for landmark training (EM residents: 4.6/5.0; PGY trainees: 4.1/5.0) (Table 3). All eight EM residents rated ultrasound-guided training 5/5 (SD 0), and PGY ratings were near-uniformly 5/5 (mean 5.0, SD 0.1). These ratings suggest high acceptability of the ultrasound-guided curriculum, although ceiling effects limit the ability to discriminate degrees of satisfaction or to infer superiority over landmark training satisfaction.

**Table 3:** Course satisfaction ratings

Measure	Emergency Residents	PGY Trainees
Landmark training	4.6 ± 0.5	4.1 ± 0.7
US-guided training	5.0 ± 0.0	5.0 ± 0.1

Data presented as mean ± SD on 5-point Likert scale (5 = strongly agree). Secondary outcomes are descriptive and were not inferentially tested

## Discussion

### Performance during the ultrasound-guided phase

This preliminary programme evaluation suggests that simulation-based education using a flipped classroom model may support learning of ultrasound-guided LP. EM residents demonstrated higher rubric-rated OSCE performance during the ultrasound-guided phase than the earlier landmark phase (nominally significant, uncorrected), while the efficiency measures favoured ultrasound without reaching significance and all participants reported favourable psychological outcomes. However, the sequential study design limits causal inference, and these findings should be considered preliminary evidence requiring replication in randomised studies.

The most notable finding was the 100% completion rate during the ultrasound-guided OSCE compared to 43% during the earlier landmark OSCE, a large difference in this simulation setting that did not reach statistical significance (*p* = 0.125) given the small paired sample. The ultrasound-guided phase was also associated with fewer needle passes and shorter procedure times, although neither difference reached statistical significance in this small sample. The direction of these simulation-based findings is consistent with clinical studies demonstrating benefits of ultrasound guidance in patient care [6], although our educational setting does not permit direct comparison. If confirmed in larger randomised studies, such improvements in training performance could translate to meaningful patient benefits, as more needle passes and traumatic insertions increase the risk of complications [4, 17].

### Psychological benefits across training levels

Both EM residents and PGY trainees reported significantly lower stress with ultrasound guidance, alongside a smaller, non-significant increase in confidence. Procedural anxiety is a documented barrier to skill acquisition [18], and ultrasound may reduce cognitive burden by providing real-time visual feedback, replacing the uncertainty inherent in ‘blind’ landmark-based techniques. PGY trainees reported higher baseline stress and lower confidence, yet relative improvements were comparable between groups, suggesting the intervention may benefit learners across the training spectrum.

### Effectiveness of flipped classroom and simulation-based education

Our curriculum incorporated evidence-based educational principles. The flipped classroom model allowed trainees to master theoretical content at their own pace before hands-on practice [13]. Consistent with Kolb’s experiential learning theory [10], the curriculum dedicated maximum time to deliberate practice with immediate feedback, a critical element for procedural skill acquisition [11]. The simulation environment supports ‘reflective observation’. By receiving immediate tactile feedback from simulator compliance and real-time visual confirmation from ultrasound imaging, trainees can immediately pause to evaluate their needle trajectory and analyze their performance. This structured reflection lets learners iterate safely and consolidate their understanding without patient risk, linking concrete experience and abstract conceptualisation [19]. High-fidelity simulation addressed the ethical tension between trainee learning and patient safety, allowing trainees to practise and receive correction without patient harm [11]. The custom 3D-printed simulator provided anatomically accurate structures, realistic ultrasound properties, and CSF aspiration feedback at an estimated material cost of USD 40 per unit compared to approximately USD 1,000 for comparable commercial lumbar puncture trainers potentially facilitating adoption in resource-limited settings. The flipped classroom model was the instructional delivery method for both training phases; this study did not compare flipped classroom versus traditional didactic approaches, and therefore cannot draw conclusions about the relative effectiveness of these delivery methods. The utility of low-cost, 3D-printed models in point-of-care ultrasound training has been increasingly recognized as a cost-effective alternative to commercial models, reducing financial barriers in medical education [20]. 3D printing has also been applied to more complex scenarios, such as scoliotic spines, indicating its broader applicability to ultrasound-guided puncture simulation [21].

### Limitations

Several important limitations must be considered. First, the sequential, non-randomised design introduces potential confounds including practice, maturation, and testing effects. All residents completed landmark training before ultrasound training; therefore, the better ultrasound-phase performance may partly reflect cumulative learning, familiarity with the OSCE format, or greater procedural readiness at a later stage, rather than the ultrasound-guided method alone. A randomised crossover or parallel-group design would be required to establish causal superiority. Second, OSCE assessment was not conducted for PGY trainees due to resource constraints, limiting generalisability of objective performance findings to the PGY population. Third, the sample size was small (*n* = 7 paired comparisons for primary outcomes), limiting statistical power and generalisability; large effect sizes in very small samples can be unstable. Fourth, all OSCE scoring was performed by a single unblinded assessor who also delivered the curriculum, so inter-rater reliability could not be assessed and the subjective overall performance rating may be inflated; the objective primary outcomes (completion status, puncture count, procedure time) are less susceptible to this bias. Fifth, psychological outcomes relied on non-validated single-item measures and should be interpreted as exploratory. We evaluated simulation performance rather than clinical outcomes, studied participants from a single institution, and did not assess long-term skill retention.

### Implications and future directions

Despite these limitations, our findings provide preliminary support for the feasibility and acceptability of ultrasound-guided LP training using simulation-based flipped classroom methods. The curriculum structure may help inform local curriculum design, pending external validation in larger, multi-centre studies. Future research should employ randomised designs with equivalent assessment conditions, include objective assessment across all trainee levels, evaluate clinical outcomes and skill retention, and conduct multi-centre studies to strengthen generalisability.

## Conclusion

This pilot programme evaluation suggests that a simulation-based flipped classroom curriculum for ultrasound-guided LP is feasible, well accepted, and associated with improved rubric-rated OSCE performance in a small sequential resident sample. Among EM residents, rubric-rated performance during the ultrasound-guided phase exceeded that during the earlier landmark phase, with nominally higher scores and ratings (uncorrected for multiple comparisons; neither difference would remain significant after correction), alongside a large but statistically non-significant increase in completion rate (100% vs. 43%, *p* = 0.125). However, the sequential design prevents definitive causal conclusions. These preliminary findings provide motivation for future randomised studies with larger samples, equivalent assessment conditions, and clinical outcome assessment.

## Supplementary Information

Supplementary Material 1.

Supplementary Material 2.

## Data Availability

Data are available from the corresponding author upon reasonable request.

## Abbreviations

CI: Confidence interval
CSF: Cerebrospinal fluid
dz: Cohen’s dz (paired effect size)
EM: Emergency medicine
LP: Lumbar puncture
OSCE: Objective structured clinical examination
PGY: Post-graduate year
SD: Standard deviation
STROBE: Strengthening the reporting of observational studies in epidemiology
US: Ultrasound
