# Supplementary material for: Simulation-based ultrasound-guided lumbar puncture training using a flipped classroom: a single-arm pilot programme evaluation
Source: BMC Med Educ. 2026 Aug 1;26:1741. doi: 10.1186/s12909-026-10070-8 (PMC13717963; doi:10.1186/s12909-026-10070-8)
Supplement: Supplementary file 1 [file 12909_2026_10070_MOESM1_ESM.docx]

**Additional file 1**

**STROBE Statement checklist**

*Checklist of items that should be included in reports of observational studies. Manuscript: "Simulation-Based Ultrasound-Guided Lumbar Puncture Training Using a Flipped Classroom: A Single-Arm Pilot Programme Evaluation." Study design: prospective single-arm sequential programme evaluation (observational).*

| **Section / Item** | **No.** | **Recommendation** | **Reported in (section)** |
| --- | --- | --- | --- |
| Title and abstract | 1a | Indicate the study’s design with a commonly used term in the title or the abstract | Title (“Single-Arm Pilot Programme Evaluation”); Abstract (Methods) |
|  | 1b | Provide in the abstract an informative and balanced summary of what was done and what was found | Abstract (Introduction/Methods/Results/Conclusion) |
| **INTRODUCTION** |  |  |  |
| Background/rationale | 2 | Explain the scientific background and rationale for the investigation | Introduction, paragraphs 1–3 |
| Objectives | 3 | State specific objectives, including any prespecified hypotheses | Introduction, final paragraph (objective + hypotheses) |
| **METHODS** |  |  |  |
| Study design | 4 | Present key elements of study design early in the paper | Methods §2.1 (prospective single-arm sequential programme evaluation) |
| Setting | 5 | Describe the setting, locations, and relevant dates (recruitment, exposure, follow-up, data collection) | Methods §2.1 (CSMUH, central Taiwan, January–June 2022) |
| Participants | 6a | Give the eligibility criteria, and the sources and methods of selection of participants | Methods §2.2 (EM residents – mandatory; PGY trainees – voluntary); Figure 1 |
|  | 6b | (Cohort/matched) matching criteria / exposure–unexposed — not applicable (single-arm; each resident is own control across sequential phases) | Methods §2.2 (paired within-resident comparison) |
| Variables | 7 | Clearly define all outcomes, exposures, predictors, potential confounders, and effect modifiers | Methods §2.5 (primary OSCE outcomes; secondary psychological/satisfaction) |
| Data sources/measurement | 8 | For each variable, give sources of data and details of methods of assessment; comparability of methods if >1 group | Methods §2.3 (intervention), §2.4 (simulator), §2.5 (measures); same rubric both OSCEs except item 2 |
| Bias | 9 | Describe any efforts to address potential sources of bias | Methods §2.2 (selection bias), §2.3 (assessors non-blinded; objective anchors, dual raters); Discussion §4.4 |
| Study size | 10 | Explain how the study size was arrived at | Methods §2.6 (no a priori sample-size calculation; convenience sample of all available trainees) |
| Quantitative variables | 11 | Explain how quantitative variables were handled in the analyses | Methods §2.5–2.6 (0–12 score, 0–5 rating, puncture count, procedure time; normality tested) |
| Statistical methods | 12a | Describe all statistical methods, including those used to control for confounding | Methods §2.6 (paired t-tests; Fisher’s exact; Cohen’s d; secondary outcomes descriptive only) |
|  | 12b | Describe any methods used to examine subgroups and interactions | Methods §2.6 / Results (residents vs PGY reported separately; descriptive) |
|  | 12c | Explain how missing data were addressed | Methods §2.2 (no dropouts; one resident missing survey → n=8; denominators stated) |
|  | 12d | (Cohort) loss to follow-up — not applicable (no follow-up losses; single session) | Methods §2.2 (no dropouts) |
|  | 12e | Describe any sensitivity analyses | Methods §2.6 (Wilcoxon signed-rank sensitivity for primary continuous outcomes) |
| **RESULTS** |  |  |  |
| Participants | 13a | Report numbers at each stage (eligible, examined, confirmed eligible, included, completing, analysed) | Results §3.1; Figure 1; Methods §2.2 (22 enrolled; paired n=7; US-phase n=9; surveys 8+13) |
|  | 13b | Give reasons for non-participation at each stage | Methods §2.2 (PGY not OSCE-assessed – resource constraints; 2 residents joined late) |
|  | 13c | Consider use of a flow diagram | Figure 1 (participant flow diagram) |
| Descriptive data | 14a | Give characteristics of study participants and information on exposures/potential confounders | Methods §2.2 (experience, prior LPs); Results §3.1–3.2; Tables 1–3 |
|  | 14b | Indicate number of participants with missing data for each variable | Methods §2.2 (n=8 residents for surveys; denominators per outcome) |
| Outcome data | 15 | Report numbers of outcome events or summary measures | Results §3.1 (OSCE); Tables 1–3 |
| Main results | 16a | Give unadjusted estimates and, if applicable, confounder-adjusted estimates and their precision | Results §3.1 (means ± SD, p-values, Cohen’s d); Table 1 |
|  | 16b | Report category boundaries when continuous variables were categorized | Not applicable (continuous outcomes not categorized; completion is inherently binary) |
|  | 16c | Translate relative risk into absolute risk for a meaningful time period if relevant | Not applicable (educational/simulation outcomes) |
| Other analyses | 17 | Report other analyses done (subgroups, interactions, sensitivity) | Results §3.1 (all-9 Panel B); Methods §2.6 (Wilcoxon sensitivity) |
| **DISCUSSION** |  |  |  |
| Key results | 18 | Summarise key results with reference to study objectives | Discussion §4.1 |
| Limitations | 19 | Discuss limitations, sources of bias/imprecision, direction and magnitude of bias | Discussion §4.4 (sequential design/confounding, no PGY OSCE, small n, non-blinding, single-item measures) |
| Interpretation | 20 | Give a cautious overall interpretation considering objectives, limitations, and other evidence | Discussion §4.1–4.3; Conclusion |
| Generalisability | 21 | Discuss the generalisability (external validity) of the study results | Discussion §4.4–4.5 (single institution; requires multi-centre replication) |
| **OTHER** |  |  |  |
| Funding | 22 | Give the source of funding and the role of funders | Funding statement (no specific grant) |

*Note: An Explanation and Elaboration article discusses each checklist item; STROBE checklist and the guidance are available at https://www.strobe-statement.org. Section numbers refer to the revised manuscript.*
