# Supplementary material for: Simulation-based ultrasound-guided lumbar puncture training using a flipped classroom: a single-arm pilot programme evaluation
Source: BMC Med Educ. 2026 Aug 1;26:1741. doi: 10.1186/s12909-026-10070-8 (PMC13717963; doi:10.1186/s12909-026-10070-8)
Supplement: Supplementary file 2 [file 12909_2026_10070_MOESM2_ESM.docx]

**Additional file 2**

**Flipped-classroom curriculum and e-learning module outline**

The six-month curriculum used a flipped-classroom model: trainees completed a self-paced online pre-learning module before each in-person workshop; a short pre-test confirmed engagement with the online content, and instructor-led time was reserved for hands-on deliberate practice (1:6 instructor-to-trainee ratio, immediate feedback).

**Month 1 — Landmark-method training**

- Online pre-learning module (15 minutes): LP indications, contraindications, anatomical landmarks, procedural steps, and complications.
- Pre-test assessment (to confirm engagement with the online content).
- In-person workshop (2 hours): step-by-step demonstration and hands-on practice on the Kyoto Kagaku Lumbar Puncture Simulator II M43B; 1:6 ratio; immediate feedback and deliberate practice.

**Month 3 — Landmark-method OSCE**

- Emergency-medicine residents; 8-minute time limit.
- Seven-item, 0–2 rubric (maximum 12 points) plus overall rating (0–5), number of punctures, procedure time, and completion status.

**Month 4 — Ultrasound-guided-method training**

- Online pre-learning module (20 minutes): ultrasound anatomy, image optimisation, target identification, needle-guidance techniques, and complication detection.
- Pre-test assessment.
- In-person workshop (2.5 hours), three stations: (1) pre-procedure ultrasound assessment (midline, intervertebral space, optimal depth and angle); (2) ultrasound-guided needle insertion on the custom 3D-printed simulator; (3) post-procedure assessment and complication detection.
- Equipment: portable ultrasound devices (Butterfly iQ, USA; BenQ T3300, Taiwan) and the custom 3D-printed lumbar-spine simulator; 1:6 ratio; real-time feedback.

**Month 6 — Ultrasound-guided-method OSCE**

- Emergency-medicine residents; 8-minute time limit (identical to the landmark OSCE).
- Same seven-item rubric; the two OSCE versions were identical except for the second item, which assessed the localisation step specific to each method (landmark palpation versus ultrasound-guided site identification).

**Post-training satisfaction survey (5-point Likert)**

Assessed separately for the landmark and ultrasound-guided modules: course content meeting expectations; overall effectiveness of the curriculum; usefulness of the online pre-learning; value of hands-on practice; and helpfulness of instructor feedback.
